# The color of health: how racism, segregation, and inequality affect the health and well-being of preterm infants and their families

**DOI:** 10.1038/s41390-019-0513-6

**Published:** 2019-07-29

**Authors:** Andrew F. Beck, Erika M. Edwards, Jeffrey D. Horbar, Elizabeth A. Howell, Marie C. McCormick, DeWayne M. Pursley

**Affiliations:** 1 Department of Pediatrics, University of Cincinnati College of Medicine, Cincinnati, OH USA; 2 Division of General & Community Pediatrics and Hospital Medicine, Cincinnati Children’s Hospital Medical Center, Cincinnati, OH USA; 3 Vermont Oxford Network, Burlington, VT USA; 4 Department of Pediatrics, Robert Larner, MD, College of Medicine, University of Vermont, Burlington, VT USA; 5 Department of Mathematics and Statistics, College of Engineering and Mathematical Sciences, University of Vermont, Burlington, VT USA; 6 Blavatnik Family Women’s Health Research Institute, Icahn School of Medicine at Mount Sinai, New York, NY USA; 7 Department of Obstetrics, Gynecology, and Reproductive Science, Icahn School of Medicine at Mount Sinai, New York, NY USA; 8 Department of Population Health Science and Policy, Icahn School of Medicine at Mount Sinai, New York, NY USA; 9 Department of Neonatology, Beth Israel Deaconess Medical Center, Boston, MA USA; 10 Department of Social and Behavioral Sciences, Harvard TH Chan School of Public Health, Boston, MA USA; 11 Department of Pediatrics, Harvard Medical School, Boston, MA USA

## Abstract

Racism, segregation, and inequality contribute to health outcomes and drive health disparities across the life course, including for newborn infants and their families. In this review, we address their effects on the health and well-being of newborn infants and their families with a focus on preterm birth. We discuss three causal pathways: increased risk; lower-quality care; and socioeconomic disadvantages that persist into infancy, childhood, and beyond. For each pathway, we propose specific interventions and research priorities that may remedy the adverse effects of racism, segregation, and inequality. Infants and their families will not realize the full benefit of advances in perinatal and neonatal care until we, collectively, accept our responsibility for addressing the range of determinants that shape long-term outcomes.

## Introduction

In *The Color of Law*, Richard Rothstein documents how, from Reconstruction to present day, local, state, and federal policies, regulations, and laws have been used to segregate Americans by race resulting in dramatic, persistent inequalities in social, economic, and educational opportunities.^1^ Black and other minority Americans live in poorer neighborhoods,^2,3^ attend lower-quality schools,^4,5^ and receive health care at lower-quality hospitals.^6–10^

**Fig. 1:**
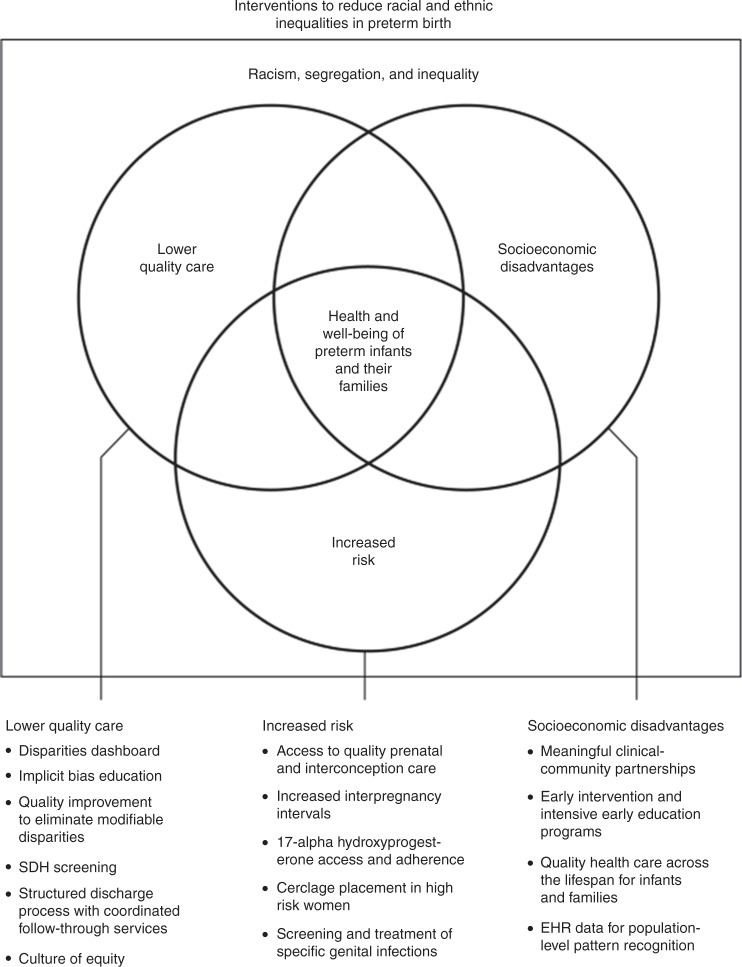
Interventions to reduce racial and ethnic inequalities in preterm birth. Three causal pathways through which racism, segregation, and inequality impact the health and well-being of preterm infants and their families with selected interventions to address each of them (more extensive list in appendix)

Racism, segregation, and inequality contribute to disparities in health outcomes across the life course.^11^ In this review, we address their effects on the health and well-being of newborn infants and their families with a focus on preterm birth. We explore three causal pathways that adversely and differentially affect outcomes for newborn infants and their families: increased risk; lower-quality care; and socioeconomic disadvantages that persist into infancy, childhood, and beyond. We propose interventions that health professionals, multidisciplinary care teams, and health-care organizations can adopt to remedy the adverse effects of racism, segregation, and inequality (Fig. 1) and research priorities to inform more effective action.

An underlying theme is that we as health professionals have the responsibility to act. The old paradigm of “follow-up” must be replaced with a new paradigm of “follow through,” the understanding that our responsibility extends beyond the hospital walls. All infants and their families will not realize the full benefit of the dramatic technical advances in perinatal and neonatal care that will occur in the 21st century unless we accept our responsibility for addressing the full range of determinants of health that ultimately shape long-term outcomes.

## Increased risk

In 2018, 10.0% of infants in the United States (U.S.) were born before 37 completed weeks’ gestation,^12^ often requiring care in a neonatal intensive care unit (NICU).^13^ Overall, 9.1% of white non-Hispanic infants were born preterm compared to 14.1% of Black non-Hispanic infants and 9.7% of Hispanic infants.^12^

Structural racism describes mutually enforcing forms of discrimination^11^ such as neighborhood deprivation,^14–16^ economic inequalities,^16,17^ educational disparities,^16,18–20^ and differential access to health care^21^ with sequelae like nutritional deficiencies^22^ and unhealthy environmental exposures.^23^ These factors are the social determinants of health (SDH), defined as the “conditions in which people are born, grow, work, live, and age.”^24^ Consistently, the SDH increase the risk for preterm birth and infant mortality.^25^

However, exposure to the SDH alone may be an insufficient explanation for increased risk for preterm birth.^26^ The early programming life course theory suggests that exposures during the perinatal period, such as maternal stress^27,28^ and depression,^29^ increase vulnerability to preterm birth. The cumulative pathway life course theory posits that additive stressors and exposures resulting in allostatic load and inflammation increase the risk for preterm birth.^30–33^ The weathering hypothesis, in which chronic stress leads to accelerated aging and earlier onset of adverse health conditions, may also be a risk factor in preterm birth.^34^

While the contribution of genetics alone likely plays a small role in differential risk for preterm birth,^35^ gene–environment interactions may explain differences.^36–39^ There is evidence of an association between DNA methylation and preterm birth among black women.^40^ Risk for preterm birth may be heritable among African-American women.^41–43^ Immigrant African women are less likely to give birth preterm than native-born black women.^44,45^ All of these findings illustrate the potential role of epigenetics in preterm birth. However, genetic and epigenetic studies must include people of diverse ancestry.^46^

Numerous experts have suggested ways to reduce differences in preterm birth rates.^47–50^ Improving and adherence to 17-alpha hydroxyprogesterone caproate after a previous preterm birth,^51^ identifying and monitoring cervical insufficiency,^28^ cerclage placement in high-risk women,^52^ screening and treatment of specific genital infections,^53,54^ and increasing interpregnancy intervals^55^ will help. Research with standardized definitions of race, ethnicity, and discrimination that seeks to disentangle the contributions of structural racism and its sequelae may inform interventions but will be difficult to conduct. Instead, research into interventions to improve access to health care, financial, and social supports for families and children, to reduce stress, and to identify and treat infections, inflammation, mental health issues, and nutritional deficiencies may have greater short-term impacts. Regardless, while broader structural issues are evaluated and being addressed, there is a clear argument for ensuring that women and their families receive the highest quality care.

## Lower-quality care

Substantial improvements in perinatal and neonatal care have led to decreasing rates of death before discharge and serious morbidities in NICUs across the U.S. Yet wide variation in mortality and serious morbidities persists.^56,57^ Black neonates die at more than twice the rate of non-Hispanic white neonates, and black and Hispanic infants remain at increased risk for severe neonatal morbidities.^58–60^ Further, the magnitude of these disparities is larger than previously reported.^61^ Disparities in severe neonatal morbidities are associated with later neurodevelopmental, behavioral, and physical impairments that disadvantage very preterm birth infants over the life course, perpetuating health and socioeconomic disparities.^59,62^

Quality of care is a crucial and potentially modifiable factor that contributes to disparities and is one of the causal pathways by which racism, segregation, and inequality adversely affect outcomes of very preterm infants. Differences in quality of care contribute to racial and ethnic disparities in two ways.^63^ First, white and minority preterm infants may receive care in different NICUs, and NICUs primarily serving minority infants may have structural characteristics associated with lower quality or have organizational models or clinical processes that lead to lower-quality care.^63^ Second, the quality of care received by preterm infants can differ by race and ethnicity in individual NICUs. These pathways may be related to organizational (e.g., culture) and clinical processes (e.g., communication) rather than structural characteristics and can confer disadvantages to minority infants. There is accumulating evidence that both mechanisms are at work.

Evidence over the past two decades has documented that black very low birth weight (VLBW) infants are cared for in a concentrated set of hospitals^64^ and that minority-serving hospitals have higher neonatal mortality rates for both black and white infants.^65,66^ More recent research has expanded beyond mortality to include severe neonatal morbidities with similar findings. Black and Hispanic very preterm infants are more likely to be born in hospitals with higher risk-adjusted neonatal morbidity and mortality rates than white VLBW infants, and disparate utilization patterns can explain 40% of the black–white disparity and nearly one third of the Hispanic–white disparity in very preterm neonatal morbidity and mortality rates.^59^ These findings have prompted investigators to evaluate the origins of outcome differences between hospitals and the potential role of differential quality of care. Investigators also found that minority infants were less likely to be born at hospitals that achieved Magnet status and infants at non-Magnet hospitals had significantly higher rates of morbidity and mortality.^67^ Similarly, VLBW infants born in hospitals that served higher proportions of minority patients had higher rates of infection, discharge without breast milk, nurse understaffing, and worse practice environments for nurses.^68^ The patient-to-nurse ratios and missed care in minority-serving hospitals were much higher than in other hospitals suggesting that improving staffing and workloads would improve quality of care at minority-serving hospitals.^69^ Recent investigations utilizing national data found significant segregation across NICUs and that black infants received care at lower-quality NICUs.^64^ These studies suggest that improving care provided at lower-quality hospitals would result in a significant narrowing of disparities.^66^

While previous studies primarily investigated between-hospital disparities, additional research has examined within-hospital disparities. Using a composite measure of quality (including both process and outcomes measures), investigators found that significant racial and ethnic variation in quality of care also existed within NICUs.^70^ Studies have highlighted within-hospital differences in the use of evidence-based practices. Hispanic mothers were found to be less likely than white mothers to receive antenatal steroids and to be feeding human milk at discharge within a given institution.^71,72^ Additional research suggests that adverse interactions with families may be a mechanism for disparate care, interactions that may be driven by clinician implicit bias and other failures in staff–family communication. These factors can affect the health of the neonate and have the potential to incite disparities that will last beyond discharge.^73^ While studies have examined the role of racism in preterm birth, few studies have examined the role of racism and bias in the NICU setting.^32^ A growing body of research in maternal health acknowledges the role that structural racism plays in generating disparities.^74^ More research on how behaviors and attitudes impact clinical outcomes in the NICU setting is needed.

There is an urgent need to go beyond describing disparities to understand their causes, and develop and implement interventions to eliminate their occurrence. An abundance of evidence demonstrates that the quality of care is one of the causal pathways by which segregation and inequality adversely affect outcomes of very preterm infants. Lessons from efforts in maternal health to reduce peripartum disparities can be applied to the NICU setting to help us further the shift from “follow-up” to “follow through.”^74,75^ Critical steps to such a shift include: enhancing communication with families by educating clinicians and staff about racial and ethnic disparities in neonatal outcomes, the importance of shared decision making, cultural competency, and assessing non-English language proficiency and providing interpreter services; education on implicit bias that can affect a clinician’s perceptions and decisions; creating disparities dashboards that stratify quality measures by race/ethnicity, insurance, language, and other attributes so that NICUs can assess how well they are taking care of infants from different backgrounds; applying quality improvement tools to narrow the disparities that are identified; engaging families and communities in quality and safety teams; and promoting a culture of equity by employing many of the tools we have used to emphasize a culture of safety. These strategies and steps must also consider, identify, and address socioeconomic disadvantages that will accompany an infant home as they are discharged from the NICU.

## Socioeconomic disadvantages in infancy and childhood

Infants of women of color begin life with greater risks of vulnerability through their well-documented increased risk for being delivered preterm and experiencing greater morbidity and mortality. In part, this disparity reflects higher parallel rates of socioeconomic disadvantage among women of color. Such disadvantage both increases the risk of preterm birth and of subsequent poorer infant health and developmental outcomes.^76,77^

Characterizing factors that underlie racial and ethnic disparities in the health and well-being among premature infants can be difficult. While the study of long-term outcomes of preterm infants has progressed over the past quarter century, much of this work has focused on simple associations between prematurity, birth weight, and NICU complications (exposures) and measures of neurodevelopment (outcomes), generally within the first few years. Because of the interest in attributing outcomes to “prematurity,” the research often employs restrictive study designs, rarely considers post-discharge factors influencing child health and development, and fails to consider variations in the quality of care on outcomes.^78^ More recent work has considered a broader range of outcomes including physical health, social and emotional well-being, and cognitive development. Studies have also examined the influence of neonatal care, illness severity, and NICU length of stay on outcomes. Nonetheless, the conceptualization of outcomes remains simplistic, failing to consider the joint effect of sociodemographic factors, chronic child illness, and maternal physical and mental health over time.

Of course, factors related to the NICU course can significantly influence physical health status in all preterm and low birth weight infants, but these infants remain vulnerable to the factors that worsen health in all children, like poverty and adverse childhood events. To the extent that infants of color are disproportionately exposed to these post-NICU stressors, they will continue to experience disparities in health outcomes of all types. Life course health development formulations suggest that the adverse effects of such stressors can be buffered by interventions that reduce the effect of stress and foster resiliency.^30^ To that effect, greater attention is now being paid to NICU-to-home transitions and to enhancing family caregiving skills via specific therapeutic and developmental interventions for the infant.

The American Academy of Pediatrics recommended guidelines for the discharge of high-risk newborns that focus on risk identification, the optimal timing of discharge, and planning for care after discharge.^79^ The guidelines emphasized discharge planning, highlighting objectives for parental education, routine health-care maintenance, management planning for unresolved medical problems, comprehensive home-care planning, identification and involvement of support services, and the determination and designation of follow-up care. These recommendations have led to greater emphasis placed on comprehensive discharge preparation programs to ensure a systematic and individualized approach for each family,^80^ including more consistent measurement, standardized discharge approaches, and the incorporation of quality improvement practices.^80–82^ As noted above, evidence suggests that some minority families experience lower-quality interactions with NICU staff, which might reduce effectiveness in discharge planning and family readiness.^73^ Deeper study of new and existing programs that support discharged preterm infants as they grow and age is also occurring.

In the years following NICU discharge, intervention programs that focus on family factors and the home environment are proving effective.^83^ Indeed, early intervention programs improve cognitive outcomes and, to a lesser extent, motor outcomes during infancy. Such intensive early educational programs are even effective for very preterm infants.^84,85^ Cognitive improvements are seen to preschool age. Those programs that focus on both the parent–infant relationship and on infant development using standardized curricula have been shown to be the most effective; however, the longer-term benefits of these programs remain to be established. Despite the evidence of their effectiveness, research has documented poorer access to such services for minority infants.^86^

An important component of how longer-term outcomes are measured is to ensure adequate characterization of the functional impact of prematurity and its complications as well as the effects of interventions. Test scores and neurologic exams may fail to provide a complete picture of child well-being. Depending on a variety of circumstances, functioning may be influenced by comorbidities (e.g., asthma, obesity, sensory deficits), movement disorders, and behavioral (e.g., attention and autism disorders) factors. To the extent that children of color experience more of these morbidities,^87^ they are at greater risk for poor functional status.

The life course models mentioned above also suggest other targets for research and intervention. Most have incorporated advances in biological, behavioral, and social science disciplines to define child development as a dynamic process that begins before conception and continues through the life span.^30^ The incorporation of emerging investigations in gene–environment interactions and epigenetic mechanisms shows promise in maternal and child health by highlighting the importance of fetal development and early childhood on the life span. These models provide an opportunity to focus on the impact of a variety of risk and protective factors in early childhood for all children and foster a shift to the promotion of more effective prevention and intervention strategies to optimize healthy development. This, in turn, informs health measurement, health-care organization, and health systems financing.^88^ It also highlights ways in which health care can meaningfully connect and collaborate with the community.

## Community interventions

NICUs are increasingly turning toward community-based prevention strategies and partnerships to improve perinatal outcomes. Strategies often precede the birth of a child, support provision of high-value care during NICU stays, and optimize transitions as patients and families return to their medical and neighborhood homes after discharge.^59,89,90^ This pivot from “follow-up” to “follow through” is accelerated by patient-level social needs screening, the use of electronic health record (EHR) data in combination with complementary datasets to support population-level pattern recognition, and bolstered clinical–community partnerships driving both patient- and population-level action.^91–96^ Many such actions elevate the relevance of SDH, which, in many ways, are thought to be “fundamental causes” of disease, factors that place individuals and populations at “risk of [having] risks.”^97^

The National Academy of Medicine and American Academy of Pediatrics, among others, now recommend routine SDH-related screening.^98,99^ They encourage routine assessment of factors like race/ethnicity, social isolation, parental educational attainment, financial strain, and residential address (to enable linkage to complementary information like neighborhood-level median household income).^98,100^ Evaluation of SDH-related risks and assets using one of the many available screening tools^101–104^ could influence clinical care from the moment of first patient/family contact, ensuring that supports are deployed to match identified needs. Assessments could start prenatally in obstetrical clinics and extend across the NICU stay to bring an awareness of context to the bedside. Indeed, families with a daily life that is characterized by racial discrimination, housing instability, food insecurity, and inflexible work schedules may have a far more difficult time adhering to complex medical regimens without additional, complementary supports.

Although screening is critical, a broader prevention mindset would benefit from strategies capable of discerning population-wide patterns in adverse health outcomes.^100,105,106^ EHR data can be leveraged to do just this, employed to inform data-driven action.^98^ For instance, EHR data can define numerators for key population health (or risk) measures. Pooling EHR data from networked birth hospitals, nurseries, NICUs, and health departments could enumerate preterm birth or infant mortality rates across geographies and in association with a range of potentially underlying sociodemographic factors. When mapped to certain neighborhoods or regions, geographic patterns could be identified. Where are “hot spots” of preterm birth and infant mortality? What are the characteristics of those areas with respect to racial segregation and socioeconomic disadvantages? By layering data elements atop one another, and by ensuring that findings are shared transparently with partners inside and outside health-care settings, new research questions and intervention strategies may be revealed.

Ultimately, the pivot from risk assessment and pattern recognition to action is what will ensure the shift from “follow-up” to “follow through.” With this as the goal, we suggest that health-care systems, including academic health centers, consider their role in population health improvement and social justice.^91,94^ Such a re-envisioned mission, one that elevates the importance of community well-being, is likely to require the added, complementary expertise that comes from meaningful community integration.^107,108^ Indeed, although health-care systems and providers have expertise in managing medical complexity, they may not always have the expertise necessary to manage social complexity. This is where clinical–community partnerships become important, valuable adjuvants. Henize et al. defined how such clinical–community partnerships can be developed, enhanced, and sustained. They described the importance of jointly defining the problem, identifying program champions, agreeing upon mutually beneficial metrics, ensuring clear and consistent communication, and planning for sustainability.^109^

The Medical–Legal Partnership (MLP) model is an example of a successful clinical–community partnership, one where legal expertise is brought into health-care settings.^110–112^ Although MLPs look different from partnership to partnership and region to region, their focus on context is consistent. For example, consider a patient in the NICU requiring ongoing respiratory and nutritional support. The intensity of their medical needs would challenge any family; that challenge is sure to be magnified by co-existent social needs. Imagine if this child were discharged to a home filled with cockroaches, a building managed by a landlord who threatened eviction with any complaint. How might this social complexity affect a family’s ability to manage the child’s medical complexity? Although health-care providers may be able to advocate for this child’s well-being, a housing attorney would amplify their voices. Through an MLP, such legal experts can advocate for remediation of adverse housing exposures and guard against illegal evictions. They may also illuminate population-level patterns.^113^ Perhaps the family from the NICU may have similarly effected neighbors. There may be other children, or expectant mothers, in the unit next door or a building located across town with the same landlord. Partnerships like MLPs work best when they extend the reach of each partner, authentically engage key stakeholders, and consistently evaluate whether they are achieving the desired outcomes.^107,114–117^

Clinical–community partnerships can extend to broader community-based investment strategies considered by health-care systems. The Healthy Neighborhood, Healthy Families Initiative in Columbus, OH involves a hospital making direct housing interventions within a “blighted” neighborhood. A multi-disciplinary team identified a racially segregated neighborhood filled with vacant housing units and characterized by high rates of poverty that was home to many of the hospital’s patients and employees. The resulting initiative involved a series of interventions focused on enhancing housing stability and quality.^116^ Although a motivated, mission-driven anchor institution galvanized the program, it coincided with the hospital assuming financial risk for community children who were insured through Medicaid. With this reality, the team suggested that investments in communities and in clinical–community partnerships, are likely to be most successful when they have both mission- and margin-oriented goals.^118^

Despite meaningful progress in screening, pattern recognition, and clinical–community partnership and action, questions clearly remain.^115^ First, the ways in which we screen warrant further assessment. When is it best to screen? What are the most relevant, important questions? For patients who remain hospitalized for months, such as those in the NICU, how frequently should screens be revisited? Second, the EHR provides us with new opportunities to consider how to document SDH-related assessments, integrate complementary data streams, and recognize population-level patterns. With these new capabilities, are there shared metrics on which obstetricians, neonatologists, and primary care pediatricians can collectively track and hold one another to account? How should metrics be shared across the clinical–community continuum (e.g., with parent advisory boards, health departments, legal aid societies, school districts, city councils)? How can we ensure that such metrics add value and are not driven by enshrined biases? Third, as we move toward action, how can we maintain ongoing stakeholder engagement, a view that stretches across the life course, and strategies that pair rigor with the reality of the real world? How can we translate this engagement across the clinical–community continuum into lasting, value-added partnerships? And finally, what is the role of the health-care system in moving toward community well-being or toward a dismantling of generations-old structural issues at the heart of unjust health outcomes? Many patients encountered in health-care settings, particularly NICUs, are medically complex. Yet their social complexity frequently determines how they ultimately experience health across the life course. As we shift our focus from “follow-up” to “follow through” for patients and populations, we must, in parallel, shift toward a more balanced approach to medical and social needs. This may require health-care systems and providers to embrace social needs screening, population-level pattern recognition, and investment in social supports and community well-being. It may also push us to identify our limitations, gaps that could be filled by willing, capable, and complementary community partners.

## Discussion

We propose three causal pathways through which racial inequality has amplified the burden of preterm birth on the health and well-being of minority communities in the U.S.: increased risk; lower-quality care; and socioeconomic disadvantages that persist into infancy, childhood, and beyond.

To improve these pathways, we must increase health equity by removing obstacles, such as discrimination, poverty, and lack of access to quality education, housing, and health care.^119^ As health-care providers, we have the obligation to “follow through,” accepting that our responsibility extends beyond the hospital walls to encompass the long-term health and well-being of the infants and families we serve. We cannot do this alone. These injustices are rooted in unconstitutional governmental actions at the local, state, and federal levels.^1^ Developing appropriate remedies must start with a national conversation that honestly confronts this history.^1,120,121^ Physicians, nurses, other health-care providers, and researchers play an important role in this conversation.

Eliminating racial disparities in preterm birth and in outcomes among premature infants and their families is an ambitious agenda that will not be accomplished quickly. Although many of the interventions identified in this review can be implemented in the near term, the sheer scope of the work is daunting, especially for hospital-based specialists and researchers who may be unfamiliar in the activities like working with community organizations or advocating for structural change. There is no tool kit of specific activities and instruments, although a list of “potentially better” practices is available in an appendix to this review. Implementation of various aspects of this agenda must be tailored to the local context of community characteristics and resources. It is not expected that health-care providers know and be responsible for all the potential services. So where to begin?

First, consider developing at least three teams. One team must address the internal culture of the health care institution and the extent to which implicit bias and other aspects of care impinge on the quality of services for all patients. Many institutions already have such teams in place; if not, models are available.^122^ A second team involves parents who can help identify the needs and potential solutions for differences in care. Principles of diversity and inclusion should be applied to this team to assure that concerns of those whose voices have long been ignored are now heard.^73,123^ The third is a community resources team with a bidirectional purpose: health-care personnel can educate community partners about the health problems and needs of premature infants and their families, and community partners can engage with health-care personnel to develop a comprehensive list of resources and services in the community and to foster communication across the hospital community. Such a group might include early intervention workers, primary care physicians, child physical and occupational therapists, social services, education services, public health professionals, community leaders, and representatives from community-based agencies.

The use of more structured data collections is encouraged. As noted above, there are now measures for screening for and assessing the SDH in clinical settings,^124,125^ a practice recommended by pediatric societies for implementation.^99,126^ Structured assessments provide assurance of more complete ascertainment of information and comparability of data over time and across units and may serve as explicit needs assessment for implementation of changes. This last step may generate the advocacy recommended above at whatever level the clinician can achieve.

We cannot wait for American society to address past injustices before we act. The interventions that we identified can be implemented today. If minority women and their infants are to fully benefit from the technical advances in perinatal and neonatal care in the 21st century, we must act now. It is our moral and professional responsibility.

## Supplementary information

Supplementary Appendix

## References

[1] Rothstein, R. *The Color of Law* (Liveright, New York, NY, 2017).

[2] Firebaugh G, Acciai F (2016). For blacks in America, the gap in neighborhood poverty has declined faster than segregation. Proc. Natl Acad. Sci. USA.

[3] Firebaugh G, Farrell CR (2016). Still large, but narrowing: The sizable decline in racial neighborhood inequality in metropolitan America, 1980-2010. Demography.

[4] Rivkin S (2016). Desegregation since the Coleman Report. Education.

[5] Kozol, J. *Savage Inequalities: Children in America’s Schools* (Broadway Paperbacks, New York, NY, 1991).

[6] Cornely PB (1956). Segregation and discrimination in medical care in the United States. Am. J. Public Health Nations Health.

[7] Reynolds PP (1997). Hospitals and civil rights, 1945-1963: the case of Simkins v Moses H. Cone Memorial Hospital. Ann. Intern. Med..

[8] Smith DB (1998). The racial segregation of hospital care revisited: Medicare discharge patterns and their implications. Am. J. Public Health.

[9] Hebert PL, Chassin MR, Howell EA (2011). The contribution of geography to black/white differences in the use of low neonatal mortality hospitals in New York City. Med. Care.

[10] Jha AK, Orav EJ, Epstein AM (2011). Low-quality, high-cost hospitals, mainly in South, care for sharply higher shares of elderly black, Hispanic, and medicaid patients. Health Aff. (Millwood).

[11] Bailey ZD (2017). Structural racism and health inequities in the USA: evidence and interventions. Lancet.

[12] Martin, J. A., Hamilton, B. E. & Osterman, M. J. K. *Births in the United States, 2018* (National Center for Health Statistics, Hyattsville, MD, 2019). 40956919

[13] Edwards, E. M. & Horbar, J. D. Variation in use by NICU types in the United States. *Pediatrics*, **142**, e20180457 (2018). 10.1542/peds.2018-0457 30282782

[14] Mehra R, Boyd LM, Ickovics JR (2017). Racial residential segregation and adverse birth outcomes: a systematic review and meta-analysis. Soc. Sci. Med..

[15] Ncube CN, Enquobahrie DA, Albert SM, Herrick AL, Burke JG (2016). Association of neighborhood context with offspring risk of preterm birth and low birthweight: a systematic review and meta-analysis of population-based studies. Soc. Sci. Med..

[16] Blumenshine P, Egerter S, Barclay CJ, Cubbin C, Braveman PA (2010). Socioeconomic disparities in adverse birth outcomes: a systematic review. Am. J. Prev. Med..

[17] Wallace ME, Mendola P, Chen Z, Hwang BS, Grantz KL (2016). Preterm birth in the context of increasing income inequality. Matern. Child Health J..

[18] El-Sayed AM, Galea S (2012). Temporal changes in socioeconomic influences on health: maternal education and preterm birth. Am. J. Public Health.

[19] Ruiz M (2015). Mother’s education and the risk of preterm and small for gestational age birth: a DRIVERS meta-analysis of 12 European cohorts. J. Epidemiol. Community Health.

[20] Blumenshine PM, Egerter SA, Libet ML, Braveman PA (2011). Father’s education: an independent marker of risk for preterm birth. Matern. Child Health J..

[21] Shapiro-Mendoza CK (2016). CDC grand rounds: public health strategies to prevent preterm birth. MMWR Morb. Mortal. Wkly. Rep..

[22] Dunlop AL, Kramer MR, Hogue CJ, Menon R, Ramakrishan U (2011). Racial disparities in preterm birth: an overview of the potential role of nutrient deficiencies. Acta Obstet. Gynecol. Scand..

[23] Burris HH, Hacker MR (2017). Birth outcome racial disparities: a result of intersecting social and environmental factors. Semin. Perinatol..

[24] Social Determinants of Health. World Health Organization http://www.who.int/social_determinants/en/ (2015). Accessed 25 May 2015.

[25] Wallace M, Crear-Perry J, Richardson L, Tarver M, Theall K (2017). Separate and unequal: structural racism and infant mortality in the US. Health Place.

[26] Mutambudzi M, Meyer JD, Reisine S, Warren N (2017). A review of recent literature on materialist and psychosocial models for racial and ethnic disparities in birth outcomes in the US, 2000-2014. Ethn. Health.

[27] Rosenthal L, Lobel M (2011). Explaining racial disparities in adverse birth outcomes: unique sources of stress for Black American women. Soc. Sci. Med..

[28] Manuck TA (2015). The phenotype of spontaneous preterm birth: application of a clinical phenotyping tool. Am. J. Obstet. Gynecol..

[29] Staneva A, Bogossian F, Pritchard M, Wittkowski A (2015). The effects of maternal depression, anxiety, and perceived stress during pregnancy on preterm birth: a systematic review. Women Birth.

[30] Lu MC, Halfon N (2003). Racial and ethnic disparities in birth outcomes: a life-course perspective. Matern. Child Health J..

[31] Kramer MR, Hogue CJ, Dunlop AL, Menon R (2011). Preconceptional stress and racial disparities in preterm birth: an overview. Acta Obstet. Gynecol. Scand..

[32] Kramer MR, Hogue CR (2009). What causes racial disparities in very preterm birth? A biosocial perspective. Epidemiol. Rev..

[33] Olson DM (2015). Allostatic load and preterm birth. Int. J. Mol. Sci..

[34] Forde, A. T., Crookes, D. M., Suglia, S. F. & Demmer, R. T. The weathering hypothesis as an explanation for racial disparities in health: a systematic review. *Ann. Epidemiol*. **33**, 1.e3–18.e3 (2019). 10.1016/j.annepidem.2019.02.011 PMC10676285 30987864

[35] Burris Heather H, Lorch Scott A, Kirpalani Haresh, Pursley DeWayne M, Elovitz Michal A, Clougherty Jane E (2019). Racial disparities in preterm birth in USA: a biosensor of physical and social environmental exposures. Archives of Disease in Childhood.

[36] Strauss JF (2018). Spontaneous preterm birth: advances toward the discovery of genetic predisposition. Am. J. Obstet. Gynecol..

[37] Burris HH, Collins JW (2010). Race and preterm birth–the case for epigenetic inquiry. Ethn. Dis..

[38] Stevenson DK (2019). Understanding health disparities. J. Perinatol..

[39] Willis E, McManus P, Magallanes N, Johnson S, Majnik A (2014). Conquering racial disparities in perinatal outcomes. Clin. Perinatol..

[40] Barcelona de Mendoza V (2017). A systematic review of DNA methylation and preterm birth in African American women. Biol. Res. Nurs..

[41] Castrillio SM, Rankin KM, David RJ, Collins JW (2014). Small-for-gestational age and preterm birth across generations: a population-based study of Illinois births. Matern. Child Health J..

[42] Dorner RA, Rankin KM, Collins JW (2017). Early preterm birth across generations among whites and African-Americans: a population-based study. Matern. Child Health J..

[43] Smid MC (2017). Maternal race and intergenerational preterm birth recurrence. Am. J. Obstet. Gynecol..

[44] Howard DL, Marshall SS, Kaufman JS, Savitz DA (2006). Variations in low birth weight and preterm delivery among blacks in relation to ancestry and nativity: New York City, 1998–2002. Pediatrics.

[45] Oliver EA (2018). Preterm birth and gestational length in four race-nativity groups, including Somali Americans. Obstet. Gynecol..

[46] Sirugo G, Williams SM, Tishkoff SA (2019). The missing diversity in human genetic studies. Cell.

[47] Spong CY, Iams J, Goldenberg R, Hauck FR, Willinger M (2011). Disparities in perinatal medicine: preterm birth, stillbirth, and infant mortality. Obstet. Gynecol..

[48] Ashton DM, Lawrence HC, Adams NL, Fleischman AR (2009). Surgeon General’s conference on the prevention of preterm birth. Obstet. Gynecol..

[49] Lu MC (2010). Closing the Black-White gap in birth outcomes: a life-course approach. Ethn. Dis..

[50] Matei, A., Saccone, G., Vogel, J. P. & Armson, A. B. Primary and secondary prevention of preterm birth: a review of systematic reviews and ongoing randomized controlled trials. *Eur. J. Obstet. Gynecol. Reprod. Biol*. **236**, 224–239 (2019). 10.1016/j.ejogrb.2018.12.022 30772047

[51] Yee LM, Liu LY, Sakowicz A, Bolden JR, Miller ES (2016). Racial and ethnic disparities in use of 17-alpha hydroxyprogesterone caproate for prevention of preterm birth. Am. J. Obstet. Gynecol..

[52] Boelig RC, Berghella V (2017). Current options for mechanical prevention of preterm birth. Semin. Perinatol..

[53] Sangkomkamhang, U. S., Lumbiganon, P., Prasertcharoensuk, W. & Laopaiboon, M. Antenatal lower genital tract infection screening and treatment programs for preventing preterm delivery. *Cochrane Database Syst. Rev.* CD006178 (2015). 10.1002/14651858.CD006178.pub3 PMC8498019 25922860

[54] Elovitz MA (2019). Cervicovaginal microbiota and local immune response modulate the risk of spontaneous preterm delivery. Nat. Commun..

[55] Hogue CJ, Menon R, Dunlop AL, Kramer MR (2011). Racial disparities in preterm birth rates and short inter-pregnancy interval: an overview. Acta Obstet. Gynecol. Scand..

[56] Horbar JD (2017). Variation in performance of neonatal intensive care units in the United States. JAMA Pediatr..

[57] Lorch SA (2017). A decade of improvement in neonatal intensive care: how do we continue the momentum?. JAMA Pediatr..

[58] Matthews TJ, MacDorman MF, Thoma ME (2015). Infant mortality statistics from the 2013 period linked birth/infant death data set. Natl Vital Stat. Rep..

[59] Howell EA (2018). Differences in morbidity and mortality rates in black, white, and Hispanic very preterm infants among New York City hospitals. JAMA Pediatr..

[60] Hamvas A (1996). The influence of the wider use of surfactant therapy on neonatal mortality among blacks and whites. N. Engl. J. Med..

[61] Janevic T (2018). Association of race/ethnicity with very preterm neonatal morbidities. JAMA Pediatr..

[62] Saigal S, Doyle LW (2008). An overview of mortality and sequelae of preterm birth from infancy to adulthood. Lancet.

[63] Howell EA (2008). Racial disparities in infant mortality: a quality of care perspective. Mt. Sinai J. Med..

[64] Horbar Jeffrey D., Edwards Erika M., Greenberg Lucy T., Profit Jochen, Draper David, Helkey Daniel, Lorch Scott A., Lee Henry C., Phibbs Ciaran S., Rogowski Jeannette, Gould Jeffrey B., Firebaugh Glenn (2019). Racial Segregation and Inequality in the Neonatal Intensive Care Unit for Very Low-Birth-Weight and Very Preterm Infants. JAMA Pediatrics.

[65] Morales LS (2005). Mortality among very low-birthweight infants in hospitals serving minority populations. Am. J. Public Health.

[66] Howell EA, Hebert P, Chatterjee S, Kleinman LC, Chassin MR (2008). Black/white differences in very low birth weight neonatal mortality rates among New York City hospitals. Pediatrics.

[67] Lake ET (2012). Association between hospital recognition for nursing excellence and outcomes of very low-birth-weight infants. JAMA.

[68] Lake ET (2015). Disparities in perinatal quality outcomes for very low birth weight infants in neonatal intensive care. Health Serv. Res..

[69] Lake ET, Staiger D, Edwards EM, Smith JG, Rogowski JA (2018). Nursing care disparities in neonatal intensive care units. Health Serv. Res..

[70] Profit Jochen, Gould Jeffrey B., Bennett Mihoko, Goldstein Benjamin A., Draper David, Phibbs Ciaran S., Lee Henry C. (2017). Racial/Ethnic Disparity in NICU Quality of Care Delivery. Pediatrics.

[71] Lee HC, Lyndon A, Blumenfeld YJ, Dudley RA, Gould JB (2011). Antenatal steroid administration for premature neonates in California. Obstet. Gynecol..

[72] Lee HC, Gould JB (2009). Factors influencing breast milk versus formula feeding at discharge for very low birth weight infants in California. J. Pediatr..

[73] Sigurdson K, Morton C, Mitchell B, Profit J (2018). Disparities in NICU quality of care: a qualitative study of family and clinician accounts. J. Perinatol..

[74] Howell EA (2018). Reduction of peripartum racial and ethnic disparities: a conceptual framework and maternal safety consensus bundle. Obstet. Gynecol..

[75] Howell Elizabeth A., Hebert Paul L., Zeitlin Jennifer (2019). Racial Segregation and Inequality of Care in Neonatal Intensive Care Units Is Unacceptable. JAMA Pediatrics.

[76] Braveman PA (2015). The role of socioeconomic factors in black-white disparities in preterm birth. Am. J. Public Health.

[77] Lorenz JM, Ananth CV, Polin RA, D’Alton ME (2016). Infant mortality in the United States. J. Perinatol..

[78] McCormick MC (1997). The outcomes of very low birth weight infants: are we asking the right questions?. Pediatrics.

[79] American Academy of Pediatrics Committee on Fetus and Newborn. (2008). Hospital discharge of the high-risk neonate. Pediatrics.

[80] Smith VC, Hwang SS, Dukhovny D, Young S, Pursley DM (2013). Neonatal intensive care unit discharge preparation, family readiness and infant outcomes: connecting the dots. J. Perinatol..

[81] Smith VC, Young S, Pursley DM, McCormick MC, Zupancic JA (2009). Are families prepared for discharge from the NICU?. J. Perinatol..

[82] Gupta, M., Pursley, D. M. & Smith, V. C. Preparing for discharge from the neonatal intensive care unit. *Pediatrics* **143**, e20182915 (2019). 10.1542/peds.2018-2915 31053622

[83] Spittle, A., Orton, J., Anderson, P. J., Boyd, R. & Doyle, L. W. Early developmental intervention programmes provided post hospital discharge to prevent motor and cognitive impairment in preterm infants. *Cochrane Database Syst. Rev.* CD005495 (2015). 10.1002/14651858.CD005495.pub4 PMC8612699 26597166

[84] The Infant Health and Development Program. (1990). Enhancing the outcomes of low-birth-weight, premature infants. A multisite, randomized trial. JAMA.

[85] McCormick MC, McCarton C, Tonascia J, Brooks-Gunn J (1993). Early educational intervention for very low birth weight infants: results from the Infant Health and Development Program. J. Pediatr..

[86] McManus B, McCormick MC, Acevedo-Garcia D, Ganz M, Hauser-Cram P (2009). The effect of state early intervention eligibility policy on participation among a cohort of young CSHCN. Pediatrics.

[87] Bauman LJ, Silver EJ, Stein RE (2006). Cumulative social disadvantage and child health. Pediatrics.

[88] Halfon N, Larson K, Lu M, Tullis E, Russ S (2014). Life course health development: past, present and future. Matern. Child Health J..

[89] Laugier O (2017). Influence of socioeconomic context on the rehospitalization rates of infants born preterm. J. Pediatr..

[90] Pursley DM, McCormick MC (2018). Bending the arc for the extremely low gestational age newborn. Pediatr. Res..

[91] Alberti PM (2018). Communities, social justice, and academic health centers. Acad. Med..

[92] Baum FE, Begin M, Houweling TA, Taylor S (2009). Changes not for the fainthearted: reorienting health care systems toward health equity through action on the social determinants of health. Am. J. Public Health.

[93] Berwick DM, Nolan TW, Whittington J (2008). The triple aim: care, health, and cost. Health Aff..

[94] Carroll-Scott A, Henson RM, Kolker J, Purtle J (2017). The role of nonprofit hospitals in identifying and addressing health inequities in cities. Health Aff..

[95] Smitherman, H. C. Jr., Baker, R. S. & Wilson, M. R. Socially accountable academic health centers: pursuing a quadripartite mission. *Acad. Med.* **94**, 176–181 (2018). 10.1097/ACM.0000000000002486 30303815

[96] Wesson DE, Kitzman HE (2018). How academic health systems can achieve population health in vulnerable populations through value-based care: the critical importance of establishing trusted agency. Acad. Med..

[97] Link, B. G. & Phelan, J. Social conditions as fundamental causes of disease. *J. Health Soc. Behav*. Spec No: 80–94 (1995). 7560851

[98] Adler NE, Stead WW (2015). Patients in context–EHR capture of social and behavioral determinants of health. N. Engl. J. Med..

[99] American Academy of Pediatrics. (2013). Community pediatrics: navigating the intersection of medicine, public health, and social determinants of children’s health. Pediatrics.

[100] Beck AF, Sandel MT, Ryan PH, Kahn RS (2017). Mapping neighborhood health geomarkers to clinical care decisions to promote equity in child health. Health Aff..

[101] Chung EK (2016). Screening for social determinants of health among children and families living in poverty: a guide for clinicians. Curr. Probl. Pediatr. Adolesc. Health Care.

[102] Fierman AH (2016). Redesigning health care practices to address childhood poverty. Acad. Pediatr..

[103] Beck AF (2016). Determinants of health and pediatric primary care practices. Pediatrics.

[104] Beck AF, Klein MD, Kahn RS (2012). Identifying social risk via a clinical social history embedded in the electronic health record. Clin. Pediatr..

[105] Hardt NS, Muhamed S, Das R, Estrella R, Roth J (2013). Neighborhood-level hot spot maps to inform delivery of primary care and allocation of social resources. Perm. J..

[106] Miranda ML, Ferranti J, Strauss B, Neelon B, Califf RM (2013). Geographic health information systems: a platform to support the ‘triple aim’. Health Aff..

[107] Kahn RS, Iyer SB, Kotagal UR (2017). Development of a child health learning network to improve population health outcomes; presented in honor of Dr Robert Haggerty. Acad. Pediatr..

[108] Wilkins CH, Alberti PM (2019). Shifting academic health centers from a culture of community service to community engagement and integration. Acad. Med..

[109] Henize AW, Beck AF, Klein MD, Adams M, Kahn RS (2015). A road map to address the social determinants of health through community collaboration. Pediatrics.

[110] Klein MD (2013). Doctors and lawyers collaborating to HeLP children–outcomes from a successful partnership between professions. J. Health Care Poor Under..

[111] Regenstein M, Trott J, Williamson A, Theiss J (2018). Addressing social determinants of health through medical-legal partnerships. Health Aff..

[112] Sandel M (2010). Medical-legal partnerships: transforming primary care by addressing the legal needs of vulnerable populations. Health Aff..

[113] Beck AF (2012). Identifying and treating a substandard housing cluster using a medical-legal partnership. Pediatrics.

[114] Sandel M (2016). Neighborhood-level interventions to improve childhood opportunity and lift children out of poverty. Acad. Pediatr..

[115] Beck AF (2018). Perspectives from the Society for Pediatric Research: interventions targeting social needs in pediatric clinical care. Pediatr. Res..

[116] Kelleher, K., Reece, J. & Sandel, M. The Healthy Neighborhood, Healthy Families Initiative. *Pediatrics* **142**, e20180261 (2018). 10.1542/peds.2018-0261 30076188

[117] Brown AF (2019). Structural interventions to reduce and eliminate health disparities. Am. J. Public Health.

[118] Sandel M, Desmond M (2017). Investing in housing for health improves both mission and margin. JAMA.

[119] Braveman PA (2019). Swimming against the tide: challenges in pursuing health equity today. Acad. Med..

[120] Coates, T. The case for reparations. The Atlantic June, 2014.

[121] Brooks, D. The case for reparations: a slow convert to the cause. The New York Times March 7, 2019.

[122] Elsesser, K. Is this the answer to diversity and inclusion? Forbes (2019).

[123] Celenza JF, Zayack D, Buus-Frank ME, Horbar JD (2017). Family involvement in quality improvement: from bedside advocate to system advisor. Clin. Perinatol..

[124] Andermann A (2018). Screening for social determinants of health in clinical care: moving from the margins to the mainstream. Public Health Rev..

[125] Acevedo-Garcia D (2014). The child opportunity index: improving collaboration between community development and public health. Health Aff..

[126] Morgenlander Marcia A., Tyrrell Hollyce, Garfunkel Lynn C., Serwint Janet R., Steiner Michael J., Schilling Samantha (2019). Screening for Social Determinants of Health in Pediatric Resident Continuity Clinic. Academic Pediatrics.

