# Supplementary material for: The color of health: how racism, segregation, and inequality affect the health and well-being of preterm infants and their families
Source: Pediatr Res. 2019 Jul 29;87(2):227–34. doi: 10.1038/s41390-019-0513-6 (PMC6960093; doi:10.1038/s41390-019-0513-6)
Supplement: Supplementary file 1 — Supplementary Appendix [file 41390_2019_513_MOESM1_ESM.docx]

**Appendix: Vermont Oxford Network Potentially Better Practices* for Follow Through**

**I. Identify social risks of NICU families and provide interventions to prevent and mitigate those risks**

1. Screen all families for social risks and social support using a standardized tool
2. Include a social worker or other social health professional on the NICU team
3. Create alliances with community organizations (clinical-community partnerships)
4. Include a paralegal or attorney on the NICU team
5. Provide parenting and family support tailored to individual family strengths and needs
6. Provide mental health services for families during the NICU stay
7. Provide referrals for drugs, alcohol, and smoking cessation counselling and treatment
8. Provide housing, meals, and transportation for NICU families
9. Provide sibling care for NICU families
10. Practice family integrated care tailored to the capabilities and needs of families
11. Provide trauma-informed care
12. Assess eligibility for SSI and other public benefits
13. Provide language support and culturally appropriate translation services for NICU families
14. Provide training and education in the social determinants of health to NICU staff
15. Provide cultural sensitivity training to NICU staff
16. Educate health care providers on implicit bias
17. Promote a culture of equity
18. Use SDH focused electronic health records to identify patterns and inform clinical decisions
19. Create a NICU culture committed to follow through

**II A. Recognize that our responsibility to families does not end at NICU discharge (transition to home)**

1. Provide discharge education and planning tailored to each family’s needs
2. Connect families with appropriate community organizations and services
3. Provide back to sleep education
4. Screen for developmental risk
5. Begin discharge planning and teaching at admission
6. Estimate discharge date at admission and revise regularly during the stay
7. Establish effective communications with the primary care provider
8. Conduct home visit before discharge and at intervals after discharge
9. Facilitate parent support groups that extend beyond the NICU stay
10. Implement strategies to identify and minimize risk for readmission
11. Use technology and social media to support families
12. Facilitate access to all necessary clinical specialists after discharge
13. Provide reminders and support services to facilitate medical visits
14. Provide mental health and addiction services for families after the NICU stay
15. Provide family planning education and contraception referral
16. Develop meaningful clinical-community partnerships

**II B. Recognize that our responsibility to families does not end at NICU discharge (Infancy)**

1. Use parent coaches to support families
2. Provide evidence based early intervention programs
3. Utilize various types and combinations of providers in the immediate transition period and subsequent visits
4. Use group visits format for families with similar issues
5. Establish a reach out and read program for patients and siblings
6. Provide medical developmental follow up
7. Establish partnerships with pre-K programs for patients and siblings
8. Provide resources regarding available public benefits
9. Develop and support tools that utilize parent reported outcomes
10. Provide family planning education and contraception referral
11. Provide access to quality high risk obstetrical care

**III. Develop robust quality improvement efforts to ensure equitable, high-quality NICU care to all newborns by eliminating modifiable disparities**

1. Establish SMART aims related to social determinants of health
2. Adopt standardized measures for social determinants of health
3. Implement a disparities dashboard
4. Develop strategies to support QI participation by economically challenged families
5. Develop strategies to support QI participation by non-traditional families
6. Develop strategies to support QI participation by racially and ethnically diverse families
7. Establish a charter with organizational leaders setting goals and resources for family advisors
8. Provide salary support for family advisors

**IV. Advocate for social justice at the local, state, and national levels**

1. Conduct and disseminate research that identifies disparities in access and outcomes
2. Serve on committees and in leadership roles within the local health system
3. Educate organizational leaders about social determinants of health
4. Engage organizational leaders with a social determinants of health charter
5. Inform and lobby at the state and national levels
6. Engage local, state, and federal agencies with responsibilities for infants and families
7. Advocate to include population health and social justice in the organizational mission
8. Speak out!

* Vermont Oxford Network refers to improvement ideas as “potentially better practices”, PBPs, rather than “better” or “best” practices to indicate that no practice is better or best until adapted, tested, and shown to work in the local context. The above list of PBPs for follow through will serve as a starting point for teams in Vermont Oxford Network quality improvement collaboratives and will be refined based on the experiences of those teams and as evidence becomes available. Used with permission of Vermont Oxford Network.
